# Highly sensitive asymmetric and symmetric cancer sensors with ultra-high-quality factor and resolution power

**DOI:** 10.1038/s41598-023-39422-w

**Published:** 2023-07-28

**Authors:** Mahdi Sovizi, Maryam Aliannezhadi

**Affiliations:** Faculty of Physics, Semnan University, PO Box: 35195-363, Semnan, Iran

**Keywords:** Photonic crystals, Optics and photonics

## Abstract

In the paper, we proposed two new highly sensitive and compact biosensors with ultra-high-quality factors based on the 1-D binary photonic crystal (silicon/air thin layer) with a defect layer. The proposed asymmetric and symmetric biosensors have just a few periods (two to five) on both sides of the defect layer and the normal cell group (INOK) and cancer cells group (YD-10B) are considered for the studies. The effects of different parameters including silicon layer thickness, air layer thickness, defect layer thickness, substrate position, number of periods, and light incident angle are considered in the biosensor operation and the biosensors are optimized based on the sensitivity. The results demonstrate that the sensitivity and defect mode wavelength of the sensors are independent of the substrate position. However, the quality factor and FOM of the sensors significantly depend on the substrate position and they are improved significantly in the symmetric sensor (~ 37% improvement in optimum condition). Also, the high sensitivities of the sensors are maintained over a wide range of silicon and air thicknesses, which is a valuable achievement in the manufacturing process. Furthermore, the sensitivity of the optimized biosensors with a defect layer thickness of 10 microns and only two periods reaches S ~ 2811 nm/RIU which is an excellent sensitivity for an optical biosensor.

## Introduction

Cancer is a major public health problem with an increasing trend in the world^1^. Timely detection and monitoring of cancer cells during the treatment significantly affects the success of the treatment and the patient’s survival. Various cancer sensors have been proposed, among which optical sensors are portable, rapid, real-time, and highly sensitive sensors with a low detection limit and great potential for diagnosing various types of cancer. Therefore, various biosensors based on exploiting resonance, scattering, chemiluminescence, luminescence, interference, fluorescence, reflectance, and absorbance have been proposed to detect cancer in the early stage. Refractive indices of normal cells are lower than cancer cells^2^. So, this difference between cancer and normal cells can be used to identify and diagnose cancer cells. Different optical sensors including surface plasmon resonance (SPR), localized surface plasmon resonance (LSPR), and the photonic crystal (PC) are sensing based on the change in the refractive index of the sensing targets^3–8^. Also, waveguide−coupled resonators containing different resonator shapes such as simple rings, ladder-shaped, Cog-shaped, and else are another type of optical sensor that works based on the change in the refractive index of the target and can be used for sensing^9–11^.

Optical sensors have some advantages including high accuracy, non-contact measurement, fast response time or real-time detection, and a wide range of applications that introduced them as excellent sensors in different fields like robotics, automotive, aerospace, medical, and more. However, they have some disadvantages such as limited sensing ranges and being cost compared to other types of sensors, being susceptible to interference, and requiring an unobstructed line−of-sight between the sensor and the object being measured, which can limit their usage in certain applications with obstacles or complex geometries. The optical sensors based on one−dimensional photonic crystals (1-D PC) have some special advantages such as a simple production process, low-cost fabrication, flexibility in structural design, simple integration to lab-on-a-chip systems with their small footprint, small sizes, the ability to establish an array of PCs to detect different targets, the ability for real-time and non-destructive sensing, high sensitivity, and quality factor/volume, which caught the attention of researchers in the last decade^12–14^. Some more detailed information about optical cancer biosensors has been discussed in reference^15^.

Most 1-D PC sensors with high sensitivity and quality factors are designed based on exploiting the defect modes created by a defect layer in the periodic configurations. Defect modes are resonance modes that can be created by differences in the thickness or refractive index of a layer or layers in the photonic crystals^16^. These sensors exploit the shift of the defect mode wavelength due to the change of electrolyte refractive index passed through the defect layer. Defect modes formed in the photonic bandgap have a low full width at half maximum (FWHM), which leads to easier detection and produces a high-quality factor sensor. A sensor with a high-quality factor can sense small changes in the refractive index of the fluid passing through the sensor. In addition, since these defect modes are resonant modes, they have a relatively high intensity. Therefore, the mentioned advantages cause to propose the structures as a suitable candidate for cancer sensors and many researchers have suggested cancer sensors based on 1-D PC with defect layers until now.

The properties of the defect mode can be tuned using different parameters. In 2022, Alhamss et al. have considered the effect of applying magnetic fields, doping rate, and the wide of n-GaAs layer used as a defect layer in a ternary 1-D PC based on Air/(Si/Bi_4_Ge_3_O_12_/SiO_2_)^4^/defect/(/(Si/Bi_4_Ge_3_O_12_/SiO_2_)^4^/Air and they demonstrated that the thickness of the defect layer is the most important parameters in control of the defect mode properties^17^. In 2022, Biswal et al. have investigated a 1-D semiconductor ternary PC with a complex unit cell and shown that the configuration can be used for designing optical wide−band filters, omnidirectional reflectors at the infrared and terahertz regimes^18^. In 2019, Ramanujam et al. have proposed a 1-D photonic crystal with defect nanocomposite layers on either side of the defect layer and a maximum sensitivity of 43 nm/RIU has been achieved for sensing cancer cells^19^. In 2020, Nouman et al. have presented a 1-D PC including Air/(SiO_2_/PbS)^3^/defect/(SiO_2_/PbS)^3^/SiO_2_ as a brain cancer sensor and they investigated the effect of defect layer thickness on sensor operation^20^. Their results declare that the sensitivity of the brain sensor increases with increasing the defect layer thickness from o.42 to 1.68 microns. In 2021, Abohassan et al. have investigated a cancer sensor based on (ZnSe/ZnS)^N^/defect/(ZnSe/ZnS)^N^ and they found an optimum thickness of 7 μm for the defect layer^21^. Also, their results show that increasing the incidence angle from 40° to 60° leads to an increase in the sensitivity of the cancer sensor from 291 to 344 nm/RIU. However, a different trend of sensitivity change has been observed by changing the incident angle applied to the blood components biosensors base on ternary periodic layers of (CaF_2_/PtSe_2_/ZnSe)^22^. The maximum sensitivity of the ternary 1-D PC biosensor was observed using the normal incident. After that, different research groups work on different parameters of the 1-D PC cancer sensors like the material of the periodic structures, the thickness of the defect layer, the number of periods, and the incident angle to achieve better performance. In most of the work, the thickness of the defect layer is more than 4 μm^2,23,24^. In 2021, Gowda et al. have proposed a cancer sensor based on 1-D PC with a periodic layer of Germanium (Ge)/Zinc Sulphide (ZnS) on both sides of the defect layer^24^ and they have investigated the effect of increasing the number of periods (from 3 to 6) on the quality factor of the sensor. Their results demonstrated that the quality factor increases with increasing the number of periods and reaches 11,323 with six periods on both sides of defect layers. In another study, Arafa et al. have reported an ultra-sensitive cancer cells sensor (2156 to 2175 nm/RIU) with a high-quality factor (2.73 × 10^5^ to 3.25 × 10^5^) based on Air/(SiO_2_/GaAs)^5^/defect/(SiO_2_/GaAs)^5^ 1-D photonic crystal with a thickness of 8.56 micron and using the incident angle 85°^23^. After that, Daher et al. have proposed cancer sensors based on Air/(Si/SiO_2_)^N^/defect/(Si/SiO_2_)^N^ 1-D PC with period numbers of $N = 5$^2^. They reached an average sensitivity of 434.7 nm/RIU with a sensor thickness of 4.2 microns and normal incident. Then, they increase the incident angle up to 85° and the sensitivity of the sensor reaches 794.69 nm/RIU. In the next step, the thickness of the defect layer has been increased, so that the thickness of the defect layer and incident angle were set at 7.26 μm and 85° respectively, and the average sensitivity of 2400.08 nm/RIU was obtained.

It is worth mentioning that optical biosensors without using a recognition-specific element like antibodies can not recognize and detect specific targets like cancer-specific biomarkers^25^. However, they can be designed to detect specific biomarkers or molecules associated with specific cancer. Identifying and capturing specific biomarkers or proteins that are overexpressed on the surface of cancer cells can be practically possible by designing and including a recognition element, such as an antibody or aptamer, specific to a particular biomarker. When the biomarker binds to the recognition element, it induces a change in the optical properties of the sensor, such as a shift in wavelength or intensity of light. This change can then be detected and measured by the optical biosensors.

Silicon is one of the abundant elements in the universe and it is well-known as the 8th most common element in the universe and 2nd in the crust of the Earth after oxygen, which causes it to be a low-cost and available material for different applications. Also, silicon is thermally stable up to 1100 °C, and large Si wafers can be handled safely without any damage due to the hardness of silicon. Furthermore, silicon thin film can be produced easily by different methods^26–28^. So, it can be a good candidate for use in optical sensors based on 1-D PC.

In the paper, we propose two new highly sensitive, small, and compact biosensors with ultra-high-quality factors based on asymmetric and symmetric 1-D binary photonic crystals, (Si/Air)^N^/Si/defect/Si/(Air/Si)^N^, and the effect of different parameters including air, silicon, defect layer thicknesses, the number of periods, and incident angle are investigated. Then, the proposed cancer sensors are optimized based on the sensitivity in the normal incident and incident angle of 85° to achieve a highly sensitive sensor with ultra-high-quality factor and figure of merit (FOM) with a small number of periods as small as two periods on both sides of the defect layer. The calculations are done using the transfer matrix method (TMM) and FORTRAN on the normal cell group (INOK) and cancer cells group (YD-10B).

## Theory and method

**Figure 1:**
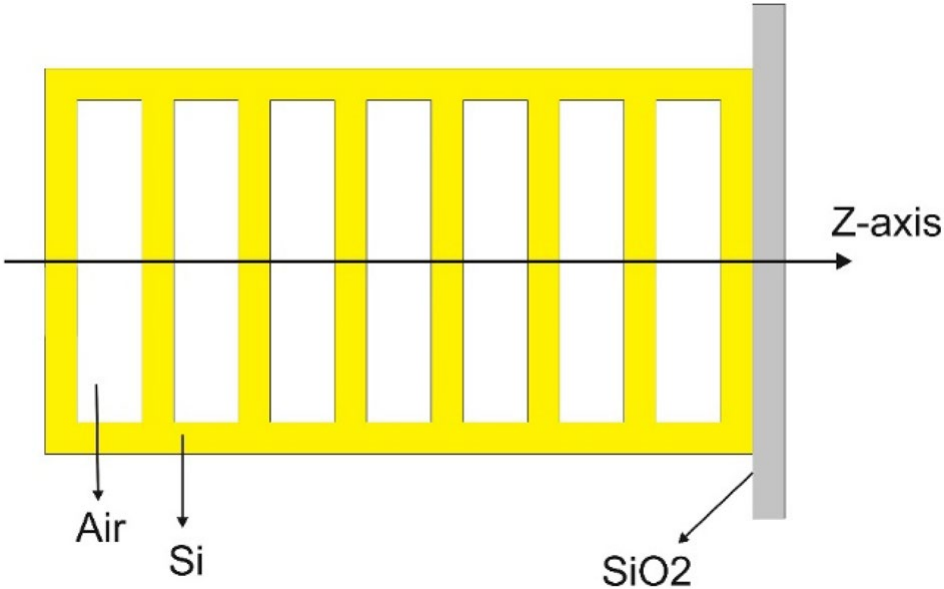
Schematic diagram of a 1-D photonic crystal consisting of alternate silicon/air layers on a silica substrate.

A schematic diagram of a 1-D photonic crystal consisting of alternate silicon/air layers on a silica substrate is shown in Fig. 1. This structure can be used as an optical sensor to detect cancer cells. For this purpose, cancerous blood or saliva can be passed through all air areas or only through the middle layer of the air, so that the cancer sensor operates according to a 1-D photonic crystal without defect or with defect, respectively.

Various numerical methods such as finite difference time domain (FDTD)^29,30^, boundary element method (BEM)^31,32^, and transfer matrix method (TMM)^12,23^ are used to analyze the optical and electromagnetic behavior of the photonic crystals. Each of these methods has some advantages. For example, BEM has a very high accuracy, which is very useful in various laser and optical problems^33–35^. TMM is also one of the most powerful, fast, and, accurate methods for modeling periodic structures like distributed feedback (DFB) lasers, distributed Bragg reflectors (DBR), and optical sensors based on photonic crystals^36–38^. The transfer matrix of each layer is as follows:

1 $$M_{j} = \left[ {\begin{array}{*{20}c} {\cos \beta_{j} } & { - i\sin \beta_{j} /p_{j} } \\ { - ip_{j} \sin \beta_{j} } & {\cos \beta_{j} } \\ \end{array} } \right],\,\,where\,\,\,p_{j} = n_{j} \cos \theta_{j}$$

where $\beta_{j} = k_{0} n_{j} d_{j} \cos \theta_{j} = 2\pi n_{j} d_{j} \cos \theta_{j} /\lambda_{o}$, $j$ represents the layer number, $d_{j}$ is the thickness of the layer $j$, $\lambda_{0}$ is the wavelength of light in air, and $\theta_{j}$ can be calculated using Snell–Descartes law:

2 $$n_{j} \sin \theta_{j} = n_{0} \sin \theta_{0}$$

where $n_{0} = 1$ and $n_{j}$ are the refractive index of air and the layer $j$, respectively. Also, $\theta_{0}$ is the initial incident angle, and $\theta_{j}$ is the incident angle in the j^th^ layer.

The transfer matrix of $m$ layers can be calculated by multiplying $M_{j}$ matrices $(j = 1,2, \ldots ,m)$ as follow:

3 $$M = \left[ {\begin{array}{*{20}c} A & B \\ C & D \\ \end{array} } \right] = M_{1} M_{2} ......M_{m}$$

where $A$, $B$, $C$, and $D$ are the matrix elements of the multilayer system.

Transmission and reflection coefficients of electric amplitudes ($t$ and $r$) and powers ($T$ and $R$) can be calculated as follow:

4 $$t = \frac{{2n_{0} }}{{An_{0} + Bn_{t} n_{0} + C + Dn_{t} }},\,\,\,\,\,T = \frac{{p_{t} }}{{p_{0} }}\left| r \right|^{2}$$

5 $$r = \frac{{An_{0} + Bn_{t} n_{0} - C - Dn_{t} }}{{An_{0} + Bn_{t} n_{0} + C + Dn_{t} }},\,\,\,\,\,R = \left| r \right|^{2}$$

**Figure 2:**
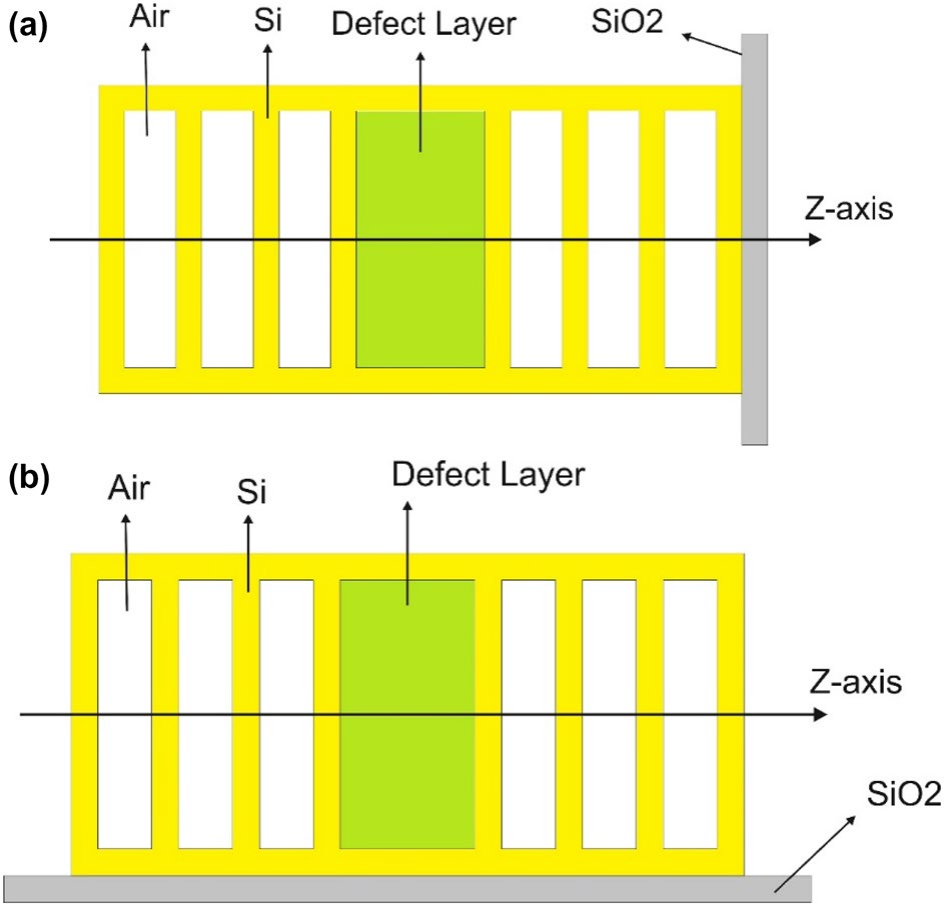
Schematic diagram of the proposed optical sensors based on 1-D photonic crystals consisting of alternate silicon/air layers. The structures are designed so that the silica substrate is (**a**) along with, and (**b**) perpendicular to the periodic structure. The proposed cancer sensors in (**a**, **b**) are labeled according to their substrate position as PC‖S (asymmetric configuration) and PC⊥S (symmetric configuration), respectively.

The transmission spectrum of perfect 1-D photonic crystals or 1-D photonic crystals without a defect layer can be exploited for sensing applications. However, this paper has no focus on these structures and is devoted to exploiting the defect modes of the proposed 1-D photonic crystals based on silicon/air layers, cancerous blood or saliva as analyte in the defect layer, and silica layer as a substrate. The silica substrate can be parallel or perpendicular to the periodic layers, which leads to passing or not passing the light through the substrate. Schematic diagrams of the proposed cancer sensors are depicted in Fig. 2. As you can observe in Fig. 2, the proposed sensors are based on a 1-D photonic crystal with a defect layer, which is located in the center of the photonic crystal, and three periods of silicon/air with an extra silicon layer are designed on both sides of the defect layer. The defect layer can be created by changing the optical properties (change the material type or refractive index) or geometrical properties (change the layer thickness) of one layer, which is almost located in the center of the structures. In fact, the proposed sensors are designed by replacing a defect layer with an air layer and changing the layer thickness. Therefore, in both proposed cancer sensors, cancerous blood or saliva are passed through the defect layer and different concentrations of cancer factors lead to the different refractive index of the defect layer and subsequently change the transmission spectrum of the structures and shift in the wavelength of the defect mode (WDM). In Fig. 2b, the position of the silica substrate is different from Fig. 2a, so that light does not pass through the substrate, which can lead to improves sensor performance. The proposed cancer sensors in Fig. 2a,b are labeled according to their substrate position as PC‖S (geometrical asymmetric configuration) and PC⊥S (geometrical symmetric configuration), respectively.

Various top–down and bottom–up methods are successfully employed by scientists to fabricate 1-D photonic crystals based on inorganic, organic, and inorganic/organic hybrid materials. The chemical vapor deposition (CVD) method, the physical vapor deposition (PVD) method, solving the precursor in appropriate solvents, and exploiting the spin coating or self-assembly have been established to produce PCs^28,39–42^. Furthermore, the formation of these 1-D PC structures is possible with the electron beam lithography (EBL) method, and similar structures have already been produced and reported with this method^43–45^. More detailed information about experimental methods for the fabrication of 1-D photonic crystals was summarized in reference^39^.

The sensitivity, quality factor, and the figure of merit (FOM) are three important quantities, which introduce to characterize the optical sensor performance. Increasing these parameters is a measure of improving the sensor performance to detect small changes in the refractive index of the target analyte. The sensitivity of the proposed sensors can be defined as the shift of defect wavelength, $\Delta \lambda_{r}$, versus the change in refractive index, $n_{d}$, of the surrounding medium (cancerous blood or saliva as analyte in the defect layer) as follows.

6 $$S = \frac{{\Delta \lambda_{r} }}{{\Delta n_{d} }}$$

Also, quality factor, *Q*, and FOM can be calculated as follows:

7 $$Q = \frac{{\lambda_{r} }}{\delta \lambda }$$

8 $$FOM = \frac{S}{\delta \lambda } = \frac{SQ}{{\lambda_{r} }}$$

where $\lambda_{r}$ and $\delta \lambda$ are the defect wavelength and the full width at half maximum (FWHM) of the defect mode.

FORTRAN and the transfer matrix method (TMM) are used to study the proposed optical cancer sensors and the results are reported in the next section. Also, it is worth mentioning that the refractive index of cancer cells can depend on various factors such as the type, size, and shape of cancer cells, the stage of cancer, and the method used to measure the refractive index. In our current study, we consider trapping a layer of cancer cells on the surface of the silicon at the boundary of the silicon-defect layer for the simulations.

## Results and discussions

**Figure 3:**
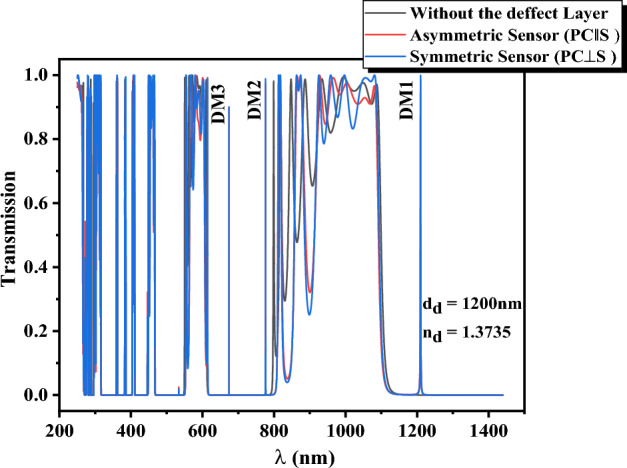
Transmission spectra of photonic crystal without defect (black solid line), PC‖S (red solid line), and PC⊥S (blue solid line) with the layer thickness of $d_{Si} = 150\,\text{nm}$, $d_{Air} = 100\,\text{nm}$, $d_{d} = 1200\,\text{nm}$, and the refractive index of the defect layer $n_{d} = 1.3735\,\text{nm}$. The photonic crystal without a defect layer consists of eight periods of silicon/air and PC‖S and PC⊥S structures have four periods of silicon/air on both sides of the defect layer.

Transmission spectra of photonic crystal without defect (black solid line) and PC‖S (red solid line) with the layer thickness of $d_{Si} = 150\,\text{nm}$, $d_{Air} = 100\,\text{nm}$, and $d_{d} = 1200\,\text{nm}$ are shown in Fig. 3 in the range of 250–1450 nm, to achieve a better understanding of the photonic bandgap positions and defect modes. For calculations, the average refractive index of this cancer cell group (YD-10B) is selected as the refractive index of the defect layer^46^, and the refractive index of the silicon layer, $n_{Si} (\lambda )$, is calculated from reference^47^ in the range of 250–1450 nm. Also, the refractive indices of air and silica layers are 1.00 and $n_{{SiO_{2} }} (\lambda = 1100nm) = 1.45$, and dispersion is neglected in the studied range for these layers. According to Fig. 3, the transmission spectrum of the 1-D photonic crystal without defect has two relatively wide photonic bandgaps in the visible region and as well as a wide photonic bandgap started from 1150 nm in the IR region. The presence of the defects layer in PC‖S (red solid line) and PC⊥S (blue solid line) results in the appearance of three localized sharp modes at the wavelengths of 1219.6, 786.3, and 674.2 nm in photonic bandgaps (PBGs) labeled as DM_1_, DM_2_, and DM_3_ in Fig. 3, respectively. These localized defect modes which are strongly dependent on the geometrical and optical properties of the defect layer and have high intensity and low FWHM, can be exploited for high-resolution sensing.

**Figure 4:**
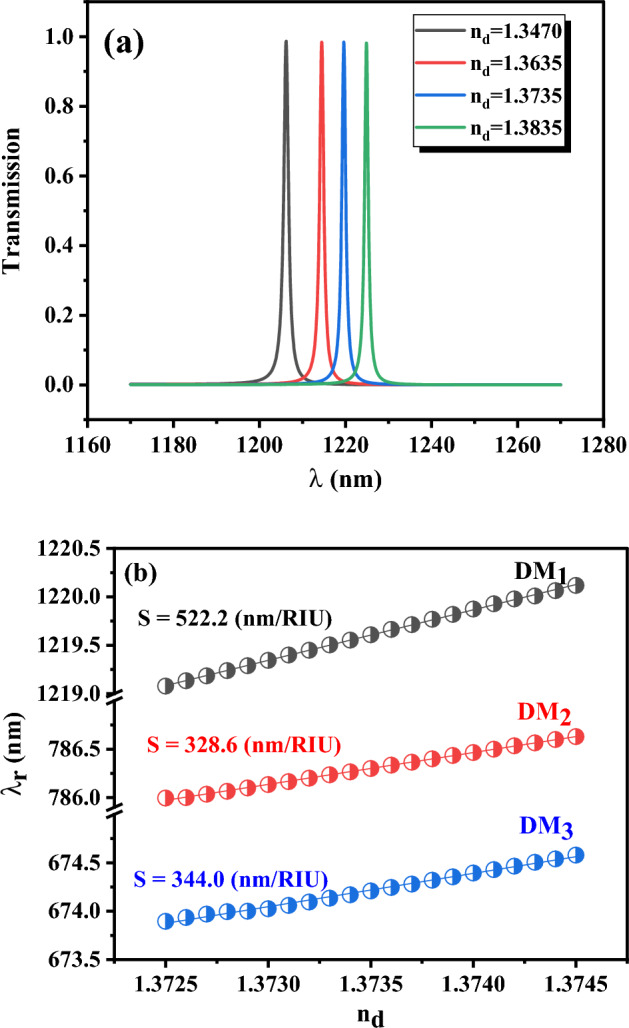
(**a**) transmission spectra of DM_1_ with four different refractive indices from the average refractive index of healthy blood (1.3470) to completely cancer blood (the YD-10B group) and (**b**) the central wavelengths of DM_1_, DM_2_, and DM_3_ versus refractive index in PC‖S sensors with the layer thickness of $d_{Si} = 150\,\text{nm}$, $d_{Air} = 100\,\text{nm}$, and $d_{d} = 1200\,\text{nm}$.

The wavelengths of localized defect modes are shifted by changing the refractive index of the analyte in the defect layer due to the presence of cancer cells. The higher value of the shift in the resonance wavelength of the defect mode by changing the analyte refractive index leads to the greater sensitivity of the optical cancer sensor, and the sensor can sense even a low change in the refractive index of cancerous blood or saliva in the defect layer. Transmission spectra of DM_1_ with four different refractive indices, from the average refractive index of healthy cells to complete cancer cells of the YD-10B cell group, are plotted in Fig. 4a to achieve a better understanding of the proposed cancer sensor operation. The results declare that replacing cancer cells with healthy cells leads to a redshift in the central wavelength of DM_1_ from 1206.2 to 1224.9 nm. Also, the central wavelengths of DM_1_, DM_2_, and DM_3_ modes versus the refractive index of cancerous blood are represented in Fig. 4b for the PC‖S sensor with the layer thickness of $d_{Si} = 150\,\text{nm}$, $d_{Air} = 100\,\text{nm}$, and $d_{d} = 1200\,\text{nm}$. The results show that increasing the refractive index causes a redshift in the central wavelength of the defect modes. The sensitivities of the PC‖S sensor for each defect mode can be obtained by fitting a linear function to each data set in Fig. 4b and it is equal to $S = 522.2\,\text{nm/RIU}$, 328.6 nm/RIU, and 344.0 nm/RIU for DM_1_, DM_2_, and DM_3_ modes, respectively.

Characteristic properties of DM_1_, DM_2_, and DM_3_ in PC‖S sensors with four periods of silicon/air layers and the layer thickness of $d_{Si} = 150\,\text{nm}$, $d_{Air} = 100\,\text{nm}$, and $d_{d} = 1200\,\text{nm}$ are calculated and gathered in Table 1 to select the appropriate defect mode for exploiting in the sensor. The refractive index of cancer cells, $n_{d} = 1.3735$, is used in the calculations of FWHM and Q factor. The sensitivities of the defect modes DM_1_, DM_2_, and DM_3_ are 522.2, 328.6, and 344.0 nm/RIU, respectively. According to the collected results in Table 1, an increase in the sensitivity and FWHM is observed with an increase in the central wavelength of the defect mode from DM_3_ to DM_1_, while the quality factor and FOM have a decreasing trend. As you can observe, the quality factor and FOM of DM_1_ are high enough for experimental work and the sensitivity of this mode for detecting cancer cells is more than DM_2_ and DM_3_ which means that DM_1_ is suitable for exploiting in the proposed cancer sensor. In addition, DM_2_ is not suitable due to its vicinity to the edge of the photonic gap, which limits the operation range of the sensor. Furthermore, the absorption of the cells at DM_1_ wavelength is significantly lower than DM_2_ and DM_3_ wavelength^48^, which is another important reason for exploiting DM_1_ for cancer cell sensing. Therefore, DM_1_ is selected for the following calculations, and geometrical optimization of the proposed optical cancer sensors is done based on the defect mode.

**Table 1:** Characteristic properties of DM_1_, DM_2_, and DM_3_ in PC‖S sensors with four periods of silicon/air layers and the layer thickness of $d_{Si} = 150\,\text{nm}$, $d_{Air} = 100\,\text{nm}$, and $d_{d} = 1200\,\text{nm}$. The Refractive index of cancer cells, $n_{d} = 1.3735$, is used as the refractive index of the defect layer for the calculation of FWHM and FOM.

	$\lambda_{r}\,\text{nm}$	$S(\,\text{nm/RIU})$	$FWHM\,\text{nm}$	$Q$	$FOM$
DM_1_	1219.608	522.21	1.071	1138.67	487.55
DM_2_	786.300	328.64	0.131	5986.75	2502.18
DM_3_	674.208	344.03	2.74e−3	246,061.16	125,556.93

### The effect of air and silicon layer thickness

**Figure 5:**
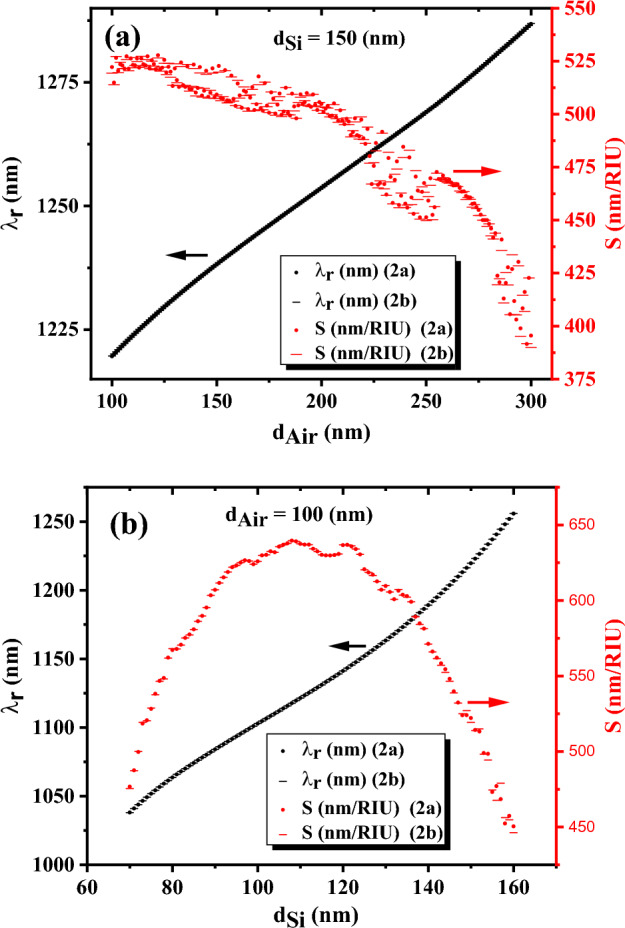
The central wavelength and sensitivity of PC‖S and PC⊥S sensors versus (**a**) the thickness of the air layer with $d_{si} = 150\,\text{nm}$, and (**b**) the thickness of the silicon layer with $d_{Air} = 100\,\text{nm}$. The thickness of the defect layer is 1200 nm and the refractive index of cancer cells, $n_{d} = 1.3735$, is used as the refractive index of the defect layer in the calculations.

In Fig. 5a, the central wavelength of DM_1_ and the sensitivity of the proposed sensors versus the thickness of the air layer are plotted. The results declare that there is no significant difference in the sensitivity and central wavelength of DM_1_ in PC‖S and PC⊥S sensors, and these quantities can be considered equal in these two proposed sensors with a good approximation. Also, the DM_1_ wavelength experiences a redshift from 1219.6 to 1286.8 nm and the sensitivity shows a decreasing trend with increasing air thickness from 100 to 300 nm. Also, in Fig. 5b, the central wavelength of DM_1_ and sensitivity of PC‖S and PC⊥S sensors versus the thickness of the silicon layer are plotted in the range of 70–160 nm. Again, almost the same sensitivity and central wavelength of DM_1_ are observed in the two proposed structures, which indicates the existence of the same optimal geometric structure based on sensitivity for PC‖S and PC⊥S sensors. Also, the central wavelength of DM_1_ experiences a redshift from 1038.0 to 1255.8 nm with increasing the silicon thickness from 70 to 160 nm. Therefore, according to Fig. 5a,b, the central wavelengths of DM_1_ in both sensors depend on the thickness of a period, $d_{Air} + d_{Si}$, in the proposed sensors, and also increasing the thickness of each layer in a period can lead to an increase in the wavelength of the defect mode. Also, the sensitivity dependences on the air and silicon layer thicknesses are different in Fig. 5a,b, and also the results demonstrate that the thickness of the air and silicon layers are two important geometric quantities in the sensor operations and they should be carefully optimized to obtain the most sensitive sensors.

**Figure 6:**
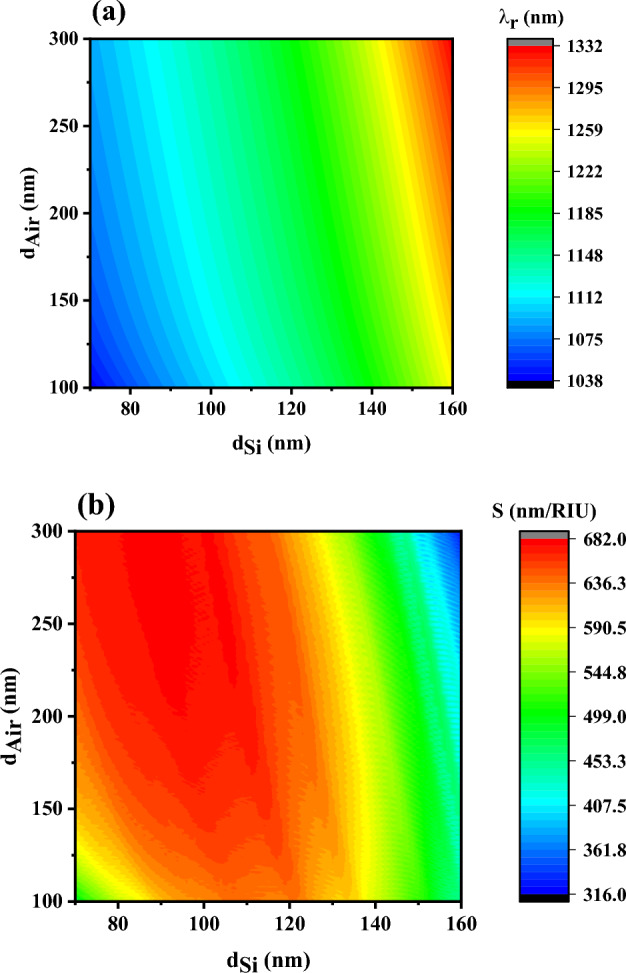
(**a**) The central wavelength, and (**b**) the sensitivity of PC‖S sensors versus the thickness of the air and silicon layers. The thickness of the defect layer is 1200 nm and the refractive index of cancer cells, $n_{d} = 1.3735$, is used as the refractive index of the defect layer in the calculations.

Now, the thicknesses of the air and silicon layers are changed simultaneously and the sensitivities of the PC‖S sensors are shown in Fig. 6b to obtain the optimum condition. Also, the calculated wavelengths of DM_1_ by simultaneously changing these two thicknesses are presented in Fig. 6a. According to the results, the wavelengths of DM_1_ depend on the thickness of a period, $d_{Air} + d_{Si}$, in the sensor and it experiences a redshift with increasing the thickness of every layer in a period. Also, increasing the thickness of silicon and air layers from 70 to 160 nm and 100 to 300 nm leads to a change in the wavelength of DM_1_ in the range of 1038 to 1332 nm, which is a significant change. Indeed, the results of Fig. 6b demonstrate that the maximum sensitivity of the PC‖S sensor (and PC⊥S sensor with a good approximation) is achieved in the range of air thickness $d_{Air} = 200 - 300\,\text{nm}$ and silicon thickness $d_{si} = 80 - 100\,\text{nm}$. Furthermore, the high sensitivity of the sensor (about $682\,\text{nm/RIU}$) is maintained over a wide range of silicon and air thicknesses. Therefore, Fig. 6a,b provide unique information for researchers and industrial owners about the appropriate thicknesses of silicon and air layers depending on the required wavelength range, and required sensor sensitivity. Also, it should be mentioned that maintaining high sensitivity over a wide range of silicon and air layer thicknesses convinces the manufacturers that small changes in the layer thicknesses during the manufacturing process lead to no significant drop in the sensitivity and performance of the proposed sensors, which is an important advantage in the manufacturing process of the sensors.

Up to now, the proposed structures were optimized based on sensitivity. However, some important quantities like FWHM, quality factor, and FOM also should be carefully investigated during the optimization process. These main characteristic parameters of the sensors are calculated for different thicknesses of the silicon and air layers and the results are presented in Table 2. According to the results, the quality factor and FOM are also significantly improved during the optimization of sensors based on sensitivity in Fig. 6b. The highest sensitivity, quality factor, and FOM of the proposed sensors are obtained in silicon and air thicknesses $d_{Si} = 89\,\text{nm}$, $d_{Air} = 275\,\text{nm}$, which are presented in the last two rows of Table 2. Therefore, during the optimization process based on sensitivity, other important quantities of the sensors are improved and these thicknesses can be introduced as the optimum geometrical conditions to achieve high-performance operation in the proposed sensors.

**Table 2:** The central wavelength of DM_1_, sensitivity, quality factor, and FOM of PC‖S and PC⊥S sensors with four periods of silicon/air layers on both sides of the defect layer, and the defect layer thickness $d_{d} = 1200\,\text{nm}$. The refractive index of cancer cells, $n_{d} = 1.3735$, is used as the refractive index of the defect layer for calculations of FWHMs and Q factors.

$$\begin{gathered} \,\,\,d_{Si} \hfill \\ (nm) \hfill \\ \end{gathered}$$	$$\begin{gathered} \,\,d_{Air} \hfill \\ (nm) \hfill \\ \end{gathered}$$	Configuration	$\lambda_{r}\,\text{nm}$	$S$ $\,\text{nm/RIU}$	$FWHM$ $\,\text{nm}$	$Q$	$FOM$
150	100	PC‖S	1219.6	522.2	1.071	1138.7	487.6
		PC⊥S	1219.7	519.4	0.933	1307.3	556.6
140		PC‖S	1189.1	571.3	0.473	2516.0	1208.8
		PC⊥S	1189.2	571.1	0.399	2979.8	1431.1
130		PC‖S	1163.4	609.6	0.247	4716.2	2471.4
		PC⊥S	1163.4	610.0	0.206	5645.7	2960.3
150	110	PC‖S	1223.8	523.3	0.881	1389.8	594.2
		PC⊥S	1223.9	521.9	0.771	1586.5	676.5
	120	PC‖S	1227.7	523.6	0.735	1670.6	712.5
		PC⊥S	1227.8	522.7	0.646	1899.7	808.7
	130	PC‖S	1231.4	521.6	0.641	1920.1	813.3
		PC⊥S	1231.4	523.8	0.568	2169.6	922.8
80	200	PC‖S	1092.4	662.9	4.26e−3	256,435.6	155,600.3
		PC⊥S	1092.4	662.9	3.48e−3	313,912.5	190,476.2
	300	PC‖S	1106.4	672.5	2.40e−3	461,002.9	280,194.8
		PC⊥S	1106.4	672.5	1.96e−3	564,493.3	343,095.7
100	200	PC‖S	1126.7	672.1	5.60e−3	201,193.2	120,025.5
		PC⊥S	1126.7	672.1	4.60e−3	244,930.9	146,118.0
	300	PC‖S	1142.0	675.8	5.66e−3	201,761.9	119,395.6
		PC⊥S	1142.0	675.8	4.66e−3	245,058.5	145,017.0
89	275	PC‖S	1118.3	681.1	2.64e−3	423,600.4	257,993.9
		PC⊥S	1118.3	681.1	2.16e−3	517,733.8	315,325.8

In addition, another significant result in Table 2 is related to FWHM, quality factor, and FOM of the defect mode in the two proposed sensors. Although the sensitivity and DM_1_ wavelength of two proposed cancer sensors with the same thicknesses of silicon and air layers are approximately equal, the FWHM and consequently quality factor and FOM of the sensors are completely different, and they are much higher in PC⊥S sensors compared with PC‖S (asymmetric optical configuration). The quality factor and FOM of PC‖S and PC⊥S sensors in optimum condition are ($Q_{\parallel } = 423{,}600.4$, $FOM_{\parallel } = 257{,}993.9$) and ($Q_{ \bot } = 517{,}733.8$, $FOM_{ \bot } = 315325.9$), respectively, which are desirable values compared to other reported sensors. This phenomenon can be understood by the high reflection (HR) resonator created by DBR on both sides of the defect layer which leads to the high confinement of the electromagnetic wave in the defect layer or analyte section. The confinement causes to increase in the photon lifetime and quality factor of the structures. Also, it leads to increasing the interaction of light with the target analyte and consequently increasing the sensitivity and FOM. Also, it should be mentioned that these values are much better in the PC⊥S sensor, and they are improved to 22% compared to in PC‖S sensor in optimum condition. The improvement is due to the change of substrate position in the PC⊥S configuration that, unlike the PC‖S sensor, light does not pass through the silica substrate.

Another important point of the proposed sensors is the high sensitivity, high-quality factor, and excellent FOM obtained in optimum geometrical conditions with only four periods on both sides of the defect layer, which is a low number of periods. It leads to reducing the size of the sensors significantly, making simplicity in the manufacturing process, and proposing them as an excellent candidate for use in compact sensors.

### The Effect of period numbers

The number of periods (2, 3, 4, and 5 periods) on both sides of the defect layer is changed and the thickness of silicon and air layers in the proposed sensors are optimized based on the sensitivity to investigate the effect of period number on the sensor’s operations. In the calculations, the thickness and refractive index of the defect layer are set on $d_{d} = 1200\,\text{nm}$ and $n_{d} = 1.3735$ (average refractive index of YD-10B cells group), respectively. The characteristic parameters of the proposed cancer sensors with different period numbers in optimum structures were calculated and the results are presented in Table 3. According to the results, the optimum sensors with different period numbers have approximately the same thicknesses of silicon and air layers. Also, the same DM_1_ wavelength and sensitivity are obtained in optimized sensors, especially after using two periods. Therefore, the sensitivity of the proposed sensors is well independent of the number of periods in the studied range. However, increasing the periods on both sides of the defect layer significantly increases the quality factor and FOM that reach up to 6,212,806 and 3,783,911, respectively, in PC⊥S with five periods. The increasing trend can be understood by increasing the reflectance of DBRs as a result of an increase in the number of periods.

**Table 3:** The central wavelength of DM_1_, sensitivity, quality factor, and FOM of PC‖S and PC⊥S sensors with an optimized thickness of silicon/air layers and different period numbers of silicon/air layers (2, 3, 4, and 5 periods) on both sides of the defect layer. The thickness and refractive index of the defect layer are $d_{d} = 1200\,\text{nm}$ and $n_{d} = 1.3735$, respectively.

Configuration	$N$	$d_{{Si}} \;{\text{(nm)}}$	$d_{{Air}} {\text{ }}\;{\text{(nm)}}$	$\lambda _{r} \;({\text{nm}})$	$S\,\text{nm/RIU}$	$FWHM\;(nm)$	$Q$	$FOM$
PC‖S	2	88	276	1116.700	681.10	0.375	2981	1818
PC⊥S				1116.705		0.306	3645	2223
PC‖S	3	89	275	1118.30		3.17e−2	35,278	21,486
PC⊥S						2.59e−2	43,111	26,257
PC‖S	4			1118.31		2.64e−3	423,600	257,994
PC⊥S						2.16e−3	517,734	315,326
PC‖S	5					2.20e−4	5,083,205	3,095,927
PC⊥S						1.80e−4	6,212,806	3,783,911

As you can observe in Table 3, the quality factor and FOM of the proposed sensors with four periods on both sides of the defect layer ($N = 4$) are high enough, so subsequent research is focused on these structures to reach an optimum sensor with easier production than five periods.

### The effect of defect layer thickness

**Figure 7:**
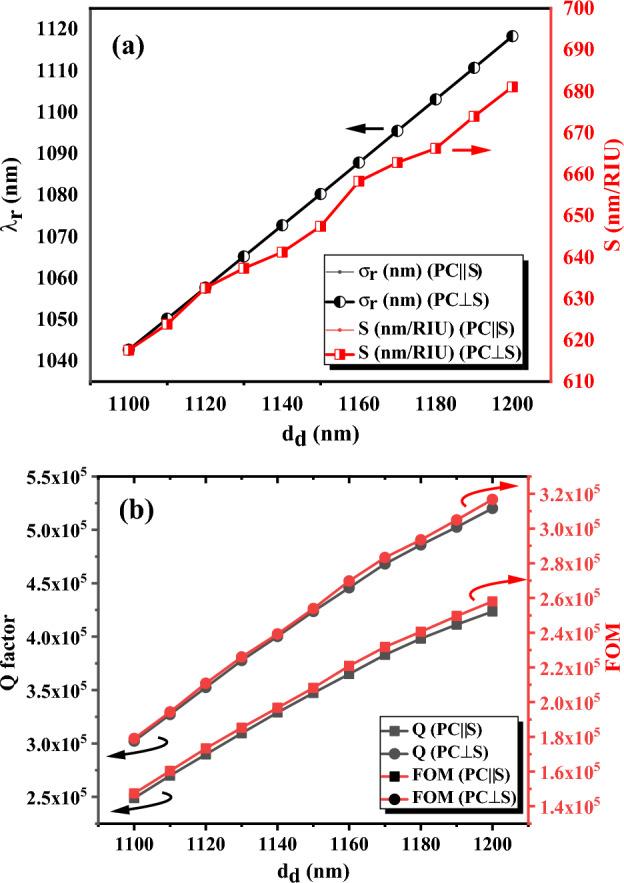
(**a**) The central wavelength and sensitivity of DM_1_, and (**b**) the quality factor and FOM of PC‖S and PC⊥S sensors with normal incident versus the thickness of the defect layer. The refractive index of cancer cells, $n_{d} = 1.3735$, is used as the refractive index of the defect layer, and four periods of silicon/air layers ($d_{Si} = 89\,\text{nm}$ and $d_{Air} = 275\,\text{nm}$) are considered in the calculations.

The thickness of the defect layer is another factor that can affect the operation of the proposed cancer sensors. However, in previous calculations, it was set at 1200 nm. In this section, the optimized cancer sensors PC‖S and PC⊥S with four periods on both sides of the defect layer are selected and the thickness of the defect layer is changed in the range of 1100 to 1200 nm to investigate the effect of the defect layer thickness on the sensor’s operations including central wavelength, sensitivity, quality factor, and FOM. The calculated central wavelength and sensitivity of the proposed sensors are shown in Fig. 7a. As you can observe, the sensitivity and DM_1_ wavelength of both sensors at different thicknesses of the defect layer are equal. Also, an increasing trend in the DM_1_ wavelength and sensitivity is observed with increasing the thickness of the defect layer. Also, the thickness of the defect layer is increased from 1.2 to 1.63 μm to investigate the effect of further increasing the thickness on sensitivity. The results declare that this increase leads to improving sensitivity from 681.1 to 855.1 nm/RIU, which means the sensitivity of the structure can be further increased with more increase in the defect layer thickness. This increasing trend of sensitivity with the defect layer thickness is consistent with other reported studies on cancer sensors based on 1-D PC^23^ and can be due to increasing the interaction of light with the target analyte infiltrated in the defect layer and consequently more change in the DM_1_ wavelength with a given change in the refractive index of the analyte.

Also, the quality factor and FOM of the sensors are plotted versus the thickness of the defect layer in Fig. 7b. The results demonstrate that increasing the thickness of the defect layer in both proposed sensors leads to an increase in the quality factor and FOM significantly. This increase can be due to increasing light and cancer cell interaction because of increasing the thickness of the defect layer. Furthermore, the quality factor and FOM of the PC⊥S sensor are remarkably better than the PC‖S sensor due to the substrate position and direction of light propagation. Therefore, increasing the thickness of the defect layer leads to improvement in the sensor operation.

### The effect of light incident angle

**Figure 8:**
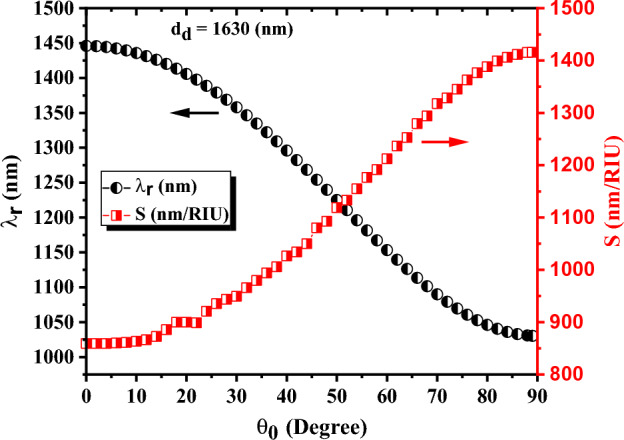
The central wavelength and sensitivity of DM_1_ versus the light incident angle in the PC⊥S sensor with four periods of silicon/air layers on both sides of the defect layer. The thicknesses of silicon, air, and defect layers are $d_{Si} = 89\,\text{nm}$, $d_{Air} = 275\,\text{nm}$, and $d_{d} = 1630\,\text{nm}$, respectively. The refractive index of cancer cells, $n_{d} = 1.3735$, is used as the refractive index of the defect layer.

The results of the previous section demonstrate that sensitivity rises with increasing the thickness of the defect layer. The light incident angle also can be another effective quantity on sensor operation that should be considered. The light incident angle is changed in the range of 0° to 89° degrees and calculated DM_1_ wavelength and sensitivity are plotted in Fig. 8. According to the results, the DM_1_ wavelength and sensitivity depend on the light incident angle significantly, and a decreasing/ increasing trend in the DM_1_ wavelength/ sensitivity is observed with increasing the light incident angle. The DM_1_ wavelength of the proposed sensor decreases from 1445.7 to 1030.4 nm and the sensitivity increases from 855.1 to 1415.8 nm/RIU (up to 65% improvement) by increasing the light incident angle from 0° to 89°, which is a significant improvement in the sensor operations. This improvement with increasing the incident angle is consistent with the other reported sensors based on 1-D PC^23,49^.

The reason for the phenomena can be explained as follows: increasing the incident angle leads to an increase in the effective thickness of the defect layer followed by an increase in the light and analyte interaction. Therefore, the sensitivity of the biosensor is improved by increasing the incident angle.

**Figure 9:**
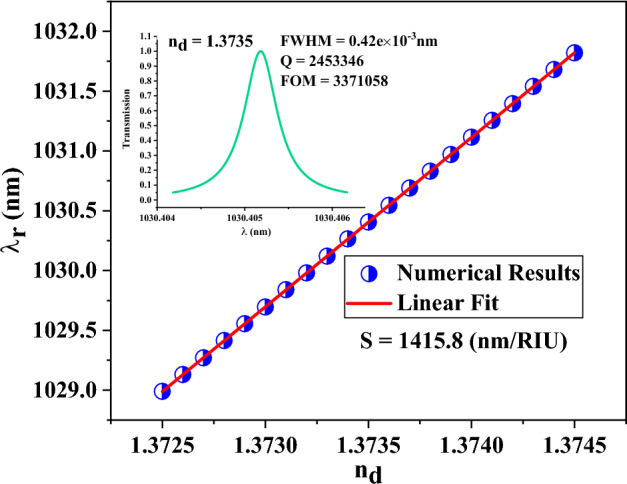
The central wavelength of DM_1_ versus refractive index in PC⊥S sensor with the layer thicknesses given in Fig. 8, the light incident angles of 89°, and only two periods on both sides of the defect layer. The transmission spectrum of the selected defect mode is plotted in the inset of the figure.

It is also worth mentioning that a high-sensitivity sensor with a small period number, even up to two periods on both sides of the defect layer, can be available by applying the light in incident angles around 89°. As depicted in Fig. 9, the sensitivity, quality factor, and FOM of the PC⊥S sensor with the given layer thicknesses of Fig. 8, the light incident angles of 89°, and only two periods on both sides of the defect layer are 1415.8 nm/RIU, 2,453,346, and 3,371,058, respectively, which are desirable values. Therefore, if there are enough facilities to apply light in high incident angles, really small sensors with a small number of periods (only two periods on both sides of the defect layer) can be produced to provide a highly sensitive sensor with lower practical limitations and smaller size for using in a compact multifunctional sensing device.

As mentioned, obtaining a low number of periods and a small highly sensitive sensor with ultra-quality factor is possible in the high incident angles. Applying the incident angle of 89° leads to the best result, but it also leads to production difficulty too. Also, the difference in the sensitivities of the sensors with the incident angles of 89° and 85° is very small (less than 6 nm/RIU), therefore, the incident angle of 85° is selected for the following investigations.

The results declare that operation parameters of the sensors including sensitivity experience a significant improvement by increasing the thickness of the defect layer and the thickness in some reported 1-D PC sensor sets at 13 times the thickness of each period^2,23^. Therefore, the thickness of the defect layer is increased up to $d_{d} = 10\mu m$, and then the biosensor is optimized based on sensitivity at the selected incident angle. The sensitivity of the optimized biosensor ($d_{Si} = 179\,\text{nm}$, $d_{Air} = 703\,\text{nm}$) increases and reaches $S = 2810.7\,\text{nm/RIU}$, which is a valuable achievement. The results of the currently reported sensors along with the current work are gathered in Table 4 to compare them. As you can observe, the proposed asymmetric and symmetric biosensors have high sensitivity and Q factor and they can be considered as a very worthy candidate for biosensing.

**Table 4:** Comparison of the sensitivity and quality factor of recently published optical biosensors along with the proposed sensors in current work.

Sensor structure	Application	Sensitivity (nm/RIU)	Q-factor	Reference
Local surface plasmon resonance	Sucrose concentration	33	–	^50^
1-D photonic crystal	Sucrose concentration	893	–	^51^
Plasmonic perfect absorber	Refractive index sensing	653	–	^52^
Photonic quasi-crystal fiber	Coronavirus	1172	–	^53^
Surface plasmon resonance	Blood glucose	2600	1500	^54^
Surface plasmon resonance	Blood glucose	1050	123.45	^55^
Surface plasmon resonance	Blood group identification	2320	–	^56^
2-D photonic crystal	HIV-1 virion	1.45 × 10^5^%/RIU	6.5 × 10^5^	^57^
Surface plasmon resonance	Biosensor	1667	–	^58^
R6G OMB DBR bio-laser	Poliovirus	1377, 0.98 × 10^4^%/RIU	4.28 × 10^3^	^59^
1-D photonic crystal	Cancerous blood cells	344	9138	^21^
1-D photonic crystal	Refractive index sensor	635	–	^60^
Asymmetric 1-D photonic crystal	Cancerous blood cells	2810.7	> 423,000	Our work
Symmetric 1-D photonic crystal	Cancerous blood cells	2810.7	> 581,000	

## Conclusion

We proposed two new portable highly sensitive and compact biosensors with ultra-high-quality factors to achieve the rapid and real-time detection of cancer cell groups. The proposed refractive index biosensors were designed based on a 1-D binary photonic crystal (silicon/air thin layer) with a defect layer (cancerous blood or saliva) to sense the low concentrations of cancer cells. The normal cell group (INOK) and cancer cell group (YD-10B) were selected for investigation. The shift of a defect mode wavelength located in the IR region with the presence of cancer cells was exploited to sense cancer cells. In the first step of studies, the order of periodic layer and defect layer thicknesses was selected with a study of the previously successful theoretical and experimental 1-D PC sensors with different materials published in scientific literature and also our previously published research in reference^12^ and a sensitivity $S = 522.2\,\text{nm/RIU}$ was achieved for the normal incident to our proposed sensors with a typical thickness of $d_{Si} = 150\,\text{nm}$, $d_{Air} = 100\,\text{nm}$, and $d_{d} = 1200\,\text{nm}$. In the second step, different parameters including the thickness of layers, the number of periods, and the incident angle were changed to achieve the optimized biosensor with the highest sensitivity, and the sensitivity of the biosensors raises and reaches S = 2810.7nm/RIU in the optimum condition ($d_{Si} = 179\,\text{nm}$, $d_{Air} = 703\,\text{nm}$), which is a valuable achievement. Therefore, it is worth mentioning that the optimized parameters of our proposed sensors (final values) are completely independent of the initial values. Furthermore, high sensitivity is maintained over a considerable range of silicon and air thicknesses, and excellent sensor performances are provided using a low number of periods, which can convince everyone that the small changes in the layer thicknesses during the manufacturing process lead to no significant drop in sensitivity and performance of the proposed cancer sensors.

## Data Availability

The datasets used and/or analyzed during the current study are available from the corresponding author upon reasonable request.
